# All-cause 2-year mortality after hospital discharge among 4273 adults in Nairobi, Kenya with a special focus on inflammatory rheumatic diseases

**DOI:** 10.7189/jogh.15.04226

**Published:** 2025-08-04

**Authors:** Benwillies Onchong’a, Tuulikki Sokka-Isler, Pekka Mäntyselkä, Ari Voutilainen

**Affiliations:** 1 University of Eastern Finland, Institute of Public Health and Clinical Nutrition, Kuopio, Finland; 2 Jasmota Hospital, Department of Rheumatology, Nairobi, Kenya; 3 University of Eastern Finland, Institute of Clinical Medicine, Kuopio, Finland; 4 Hospital Nova, Wellbeing Services County of Central Finland, Jyväskylä, Finland; 5 Kuopio University Hospital, Wellbeing Services County of North Savo, Kuopio, Finland

Findings concerning the mortality rate among inflammatory rheumatic disease (IRD) patients compared to patients without IRD indicate an increased rate amongst the former [1]. On the other hand, the difference in mortality rates appears to be somewhat negligible in countries where patients have access to modern treatments and advanced rheumatology care [2,3]. By and large, however, studies following a prospective design in this context are scarce in number [4,5].

The increased mortality risk amongst IRD patients has been associated with an increased risk of infection due to lowered immune response and drug related side effects but especially with a high level of inflammation which is associated with comorbidities, such as cardiovascular diseases and certain malignancies [6]. The chronic dysregulated systemic immune response in IRD causes damage to healthy tissues leading to widespread inflammation and damage that increases susceptibility to infections and other inflammation-associated conditions including, for example, cardiovascular diseases and haematologic cancers [7]. These complications contribute to higher mortality rates in IRD patients. Moreover, the risk of mortality differs amongst different rheumatic diseases because of underlying pathogenetic mechanisms and different disease-specific factors [5,8].

A better management of underlying disease factors and novel therapeutic options have generally contributed to reduction in the excess mortality in IRD patients [9]. This, however, is not easy to generalise globally because the quality of health care and that of scientific research are far from equal across countries.

While high-income countries have documented substantial improvements in IRD mortality over the past three decades, the evidence base from low- and middle-income countries (LMICs) remains severely limited. A South African cohort study of rheumatoid arthritis patients reported high burden of cardiometabolic comorbidities with serious infections and tuberculosis being a major cause of morbidity and mortality [10]. A cross-sectional study from Ghana reported a notable burden of disease among RA patients, potentially linked to comorbidity [11]. This shows that the role of comorbidities in driving IRD mortality appears particularly pronounced in African settings.

In high-income countries, IRD diagnosis and management typically occur within well-established rheumatology specialised services. In contrast, most public hospitals in Kenya, including major referral centres serving metropolitan areas, lack dedicated rheumatology specialised care [12]. This absence of specialised care means that IRD diagnosis and management typically fall within the scope of general internal medicine physicians, who may have limited experience in complex rheumatologic conditions. Consequently, diagnostic procedures may not fully align with international classification criteria, potentially leading to delayed diagnosis, misclassification, or suboptimal treatment initiation.

Hospital-based mortality surveillance in the Kenyan context reflects not only the history of IRD but also the cumulative impact of systemic health care limitations. Patients presenting to public hospitals often represent those with more advanced diseases, higher comorbidity burdens, or those who have experienced delays in appropriate care due to access barriers [13]. This selection bias toward more severely ill patients may contribute to higher observed mortality rates compared to population-based studies in well-resourced health care systems.

The Kenyan contextual factors are crucial for interpreting mortality outcomes and understanding the potential for intervention. While the present study documents the mortality burden among IRD patients in this setting, it also highlights the urgent need for health care system strengthening, including the development of rheumatology specialised services, improved diagnostic capabilities, and enhanced access to evidence-based treatments.

## METHODS

### Data gathering

Data were provided by Mbagathi County Hospital, one of the most frequented public hospitals in Nairobi metropolitan area, Kenya. The local health records staff identified patients from the hospital admission registry and collected individual-level data from paper-based and electronic medical records. Data concerning deaths were extracted from the Ministry of Health Inpatient and Outpatient Morbidity and Mortality Register. Kenyan National Identity Card Numbers were applied to combine the patients with their medical records.

For this study, patients were classified as IRD and non-IRD patients according to their diagnoses on the registries. Non-IRD participants were all patients that had diseases other than IRD diseases. Inflammatory rheumatic diseases referred to the following diagnoses: arthritis (psoriatic arthritis, ankylosing spondylitis, and enteropathic arthritis), n = 301; acute inflammatory arthropathy (patients experiencing acute inflammatory joint symptoms that do not align with chronic or specific IRDs but excluding *e.g*. gout, bursitis, and septic arthritis), n = 108; rheumatoid arthritis (RA, n = 39); rheumatic fever (without mention of heart involvement), n = 2; and systemic sclerosis (n = 2).

Given the absence of specialised rheumatology services at the study hospital, a systematic approach was implemented to verify IRD diagnoses and ensure diagnostic consistency. The diagnostic process followed a standardised protocol established by the hospital's internal medicine department, adapted from international guidelines but modified to accommodate available resources and local clinical expertise. All IRD diagnoses were made by qualified medical doctors following a structured clinical evaluation that included:

(i) comprehensive patient history focusing on joint symptoms, morning stiffness duration, and functional limitations;

(ii) systematic physical examination documenting joint swelling, tenderness, and range of motion limitations;

(iii) assessment of extra-articular manifestations when present;

(iv) evaluation of symptom duration to distinguish acute from chronic inflammatory conditions.

Serological testing was available but not consistently performed for all patients due to resource constraints and clinical judgment. The following laboratory tests were utilised when clinically indicated and financially accessible to patients: Rheumatoid Factor (RF) testing was available and performed in 78% of suspected RA cases; Anti-Citrullinated Protein Antibody (ACPA) testing was available but performed in only 31% of suspected RA cases due to higher cost; Antinuclear Antibody (ANA) testing was performed in 45% of patients with suspected systemic connective tissue diseases; and standard inflammatory markers (ESR, CRP) were more consistently available and performed in 89% of IRD patients. Due to limited access to specialised serological testing and advanced imaging modalities, the diagnostic approach relied more heavily on clinical presentation and basic laboratory findings compared to resource-rich settings.

To enhance diagnostic accuracy despite resource limitations, several quality assurance measures were implemented:

(i) all IRD diagnoses required documentation in medical records by attending physicians with clear justification for the diagnostic classification;

(ii) cases with ambiguous presentations were reviewed by the senior internal medicine physician before final classification;

(iii) Diagnostic codes were cross-referenced with clinical notes to ensure consistency between assigned ICD-10 codes and documented clinical findings;

(iv) patients with acute inflammatory symptoms lasting <6 weeks were classified separately as ‘acute inflammatory arthropathy’ to distinguish them from chronic IRD conditions.

At baseline, patients who were admitted to the hospital between 1 January and 30 September 2022, were considered as potential ones to be included in the study. Data regarding patient characteristics (age, height, sex, education level, income level, employment status, physical activity level, drinking status, and smoking status), the date of hospital admission, and the reason for hospitalisation were gathered. For each patient their medical records provided the primary disease for the hospitalisation together with the main comorbidity or the information that comorbidities do not exist. The baseline study cohort included 4420 patients of which 147 died during hospitalisation. In the sample size calculation, we expected the prevalence of 0.1 in IRD patients and that of 0.075 in non-IRD patients. The ratio of non-IRD to IRD patients was expected to be 3. These expectations yielded the required minimum n of 815 for IRD and that of 2443 for non-IRD patients. Onchong’a et al. report the study baseline [14].

The follow-up started when the patient was discharged after the initial hospitalisation and ended when the patient was last time admitted to the hospital or died within two years from baseline. Follow-up utilised a multi-source tracking approach. First, Kenyan National Identity Card Numbers were used to identify subsequent hospitalisations at the study hospital or affiliated facilities within the Nairobi metropolitan area. Second, monthly queries of the national Inpatient and Outpatient Morbidity and Mortality Register were conducted using patient identifiers. Third, for patients without documented subsequent contact, telephone follow-up was attempted using contact information from medical records. Loss to follow-up occurred largely due to migration outside the study area or untraceable contact information. Patients lost to follow-up were censored at their last known contact date. Comparison of baseline characteristics between patients with complete *vs*. incomplete follow-up showed no significant differences in age, sex, or IRD status, suggesting loss was random rather than systematic.

Death status was determined through multiple sources to minimise underreporting. First, deaths occurring during subsequent hospitalisations were captured through institutional mortality registers. Second, institutional death records were cross-referenced with Ministry of Health mortality databases using National Identity Card Numbers; and third, deaths reported by family members during follow-up contacts were verified through official death certificates when available. A pseudonymised data set was generated for research purposes.

The data gathering procedure as well as the use of the gathered data for research purposes were authorised and ethically approved by the Kenyan Ministry of Health Research Ethics Committee (County Government of Nairobi) on 22 August 2022 (MOH/P/78/OOK8191).

### Outcome and covariates

The vital status at the end of follow-up served as the outcome variable. In statistical analyses, it was applied as a dichotomous variable; alive or died from any cause. Consequently, the cause of death was not necessarily connected to the reason of hospitalisation.

As a routine practice, age, sex, education, income, employment, physical activity, alcohol drinking, and tobacco smoking were self-reported at the admission to the hospital and recorded in the patient files, whereas weight was measured at the hospital. Comorbidities were retrieved from medical records.

Education was categorised as Primary, Secondary, or Tertiary. In accordance with the Kenyan education system, the primary category refers to the lowest level of education between grade 1 and grade 8. The secondary category refers to the middle-level of education or high school. The tertiary category represents higher education that is obtained after secondary education or high school.

Income was reported as Low, Middle, or Upper. The categorisation is based on the Kenya Bureau of Statistics classification in which people belonging to the low-, middle-, and upper-income categories earn ≤23 670, 23 671 – 119 999, and ≥120 000 Kenyan shillings (KES) per month, respectively [15].

Employment was categorised as Formal, Informal, or Unemployed. Formal refers to having a daily job which involves a contract between the employer and employee. Informal refers to jobs with no contract between the employer and employee. Unemployment refers to having no job.

Regarding the physical activity level (PAL), the categorisation was based on recommendations from the nutrition and physiotherapy departments in the local health facility. Physical activity level was counted as the number of times the patient was involved in any form of voluntary physical exercise within one week. Less than 4 times was categorised as Low, 4 – 7 times as Moderate, and more than 7 times as High PAL. Alcohol drinking and tobacco smoking statuses were categorised as never, previous, or current.

Comorbidities were categorised according to the ICD-10.

### Statistical analysis

Descriptive analyses of baseline characteristics were conducted according to the IRD status (IRD *vs*. no IRD). Means (x̄) and standard deviations (SD) were reported for continuous variables (age and weight), n and proportions for categorical variables (sex, education, income, employment, physical activity, alcohol drinking, and tobacco smoking). The independent samples *t* test was used to test differences in the continuous variables between the IRD and non-IRD patients. Correspondingly, the Mann-Whitney U test and the Pearson χ^2^ test were used to test differences in the categorical variables.

The hierarchical Cox proportional hazard model was applied to predict the hazard of death in patients with vs without IRD. Model 1 was adjusted for age and sex, Model 2 for all baseline characteristics presented in **Table 1**. The proportional hazards assumption for the Cox model was evaluated by means of Schoenfeld residuals covariate by covariate, and no violations were detected. There were some values missing completely at random (Little’s MCAR test; *P* = 0.696) with respect to the following covariates: education (1.0%), income (2.3%), employment (1.1%), and PAL (1.2%). Missing values were not replaced to ensure data integrity.

**Table 1:** Baseline characteristics.

Characteristic*	Total	No IRD	IRD	*P*-value
n	4273	3821	452	NA
Age in years, x̄ (SD)	61.8 (10.2)	61.6 (10.3)	63.7 (8.8)	<0.001
Women weight in kg, x̄ (SD)	70.0 (9.5)	69.7 (9.7)	72.7 (7.5)	<0.001
Men weight in kg, x̄ (SD)	69.8 (9.7)	69.6 (10.0)	72.1 (7.2)	<0.001
Sex				0.826
*Women*	1964 (46.0)	1754 (45.9)	210 (46.5)	
*Men*	2307 (54.0)	2066 (54.1)	242 (53.5)	
Education level				0.085
*Primary*	673 (15.8)	608 (15.9)	65 (14.4)	
*Secondary*	2222 (52.0)	1975 (51.7)	247 (54.6)	
*Tertiary*	1334 (31.2)	1203 (31.5)	131 (29.0)	
*Unknown*	44 (1.0)	35 (0.9)	9 (2.0)	
Income level				0.399
*≤23 670 KES/mo*	1647 (38.5)	1460 (38.2)	187 (41.4)	
*23 671 − 119 999 KES/mo*	2526 (59.1)	2270 (59.4)	256 (56.6)	
*Unknown*	100 (2.3)	91 (2.4)	9 (2.0)	
Employment status				0.378
*Formal*	836 (19.6)	755 (19.8)	81 (17.9)	
*Informal*	1973 (46.2)	1756 (46.0)	217 (48.0)	
*Unemployed*	1417 (33.2)	1265 (33.1)	152 (33.6)	
*Unknown*	47 (1.1)	45 (1.2)	2 (0.4)	
Physical activity level				0.295
*Low*	1162 (27.2)	1025 (26.8)	137 (30.3)	
*Moderate*	3051 (71.4)	2744 (71.8)	307 (67.9)	
*High*	10 (0.2)	8 (0.2)	2 (0.4)	
*Unknown*	50 (1.2)	44 (1.2)	6 (1.3)	
Alcohol drinking status				<0.001
*Never drinkers*	3828 (89.6)	3452 (90.3)	376 (83.2)	
*Previous drinkers*	252 (5.9)	201 (5.3)	51 (11.3)	
*Current drinkers*	193 (4.5)	168 (4.4)	25 (5.0)	
Tobacco smoking status				0.373
*Never smokers*	3801 (89.0)	3407 (89.2)	394 (87.2)	
*Previous smokers*	153 (3.6)	136 (3.6)	17 (3.8)	
*Current smokers*	319 (7.5)	278 (7.3)	41 (9.1)	

IRD – Inflammatory Rheumatic Disease, KES – Kenyan Shilling, x̄ – mean, SD – standard deviation

*Values are presented as n (%), unless otherwise specified.

For the sensitivity analysis by the subgroups formed according to baseline characteristics, age was categorised as follows: young adulthood (<40-year-old), middle-age (40 – 60-year-old), younger-old (61 – 74-year-old), and older-old (≥75-year-old).

*P*-value <0.05 was considered as a sign of statistical significance in each analysis. IBM® SPSS® Statistics version 27 (IBM Corp., Armonk, NY, USA) was used for all statistical analyses.

## RESULTS

### Baseline characteristics

The follow-up study cohort included 4273 patients of which 452 with IRD and 3821 with no IRD. Of them, 210 (47%) of IRD and 1754 (46%) of non-IRD patients were women (**Table 1**). Patients with IRD were older (mean age 64 *vs*. 62 years, *P* < 0.001), heavier (mean weight 72 *vs*. 70 kg, *P* < 0.001), and more often alcohol drinkers (17 *vs*. 10%, *P* < 0.001). No differences were detected in other baseline characteristics between IRD and non-IRD patients (**Table 1**).

According to ICD-10 diagnose blocks the most common reason other than IRD for the baseline hospitalisation was a disease of the digestive system (n = 1478, 35%) followed by a disease of the circulatory system (n = 716, 17%), and a disease of the musculoskeletal system and connective tissue, excluding IRDs (n = 432, 10%) (**Table 2**). The three most common diseases of the digestive system were inflammatory bowel disease including ulcerative colitis (n = 327), peptic ulcer disease (n = 146), and antibiotic associated colitis (n = 132). In the category of circulatory system diseases, the most common conditions were coronary heart disease (n = 368) and hypertension (n = 96). Correspondingly, osteoporosis (n = 129) and osteomyelitis (n = 88) were the two most common musculoskeletal and connective tissue diseases, excluding IRDs.

**Table 2:** All-cause mortality rate during the 2-y follow-up period by the reason for baseline hospitalisation

Comorbidity*	n (%)	Deaths (n)	Rate (%)
Inflammatory Rheumatic Disease	452 (11)	73	16
ICD-10 diagnosis A (Septicemia, mainly)	19 (0.4)	<10	N/A
ICD-10 diagnosis B (Hepatitis A, mainly)	193 (4.5)	12	6.2
ICD-10 diagnosis D (Lipoma)	86 (2.0)	11	13
ICD-10 diagnosis E (Endocrine, nutritional, or metabolic)	35 (0.8)	<10	N/A
ICD-10 diagnoses E and M (Diabetes mellitus and gout)	13 (0.3)	<10	N/A
ICD-10 diagnosis G (Multiple sclerosis, mainly)	121 (2.8)	19	16
ICD-10 diagnosis I (Circulatory system)	716 (17)	80	11
ICD-10 diagnosis J (Respiratory system)	248 (5.8)	26	10
ICD-10 diagnosis K (Digestive system)	1478 (35)	179	12
ICD-10 diagnosis L (Psoriasis or urticaria)	136 (3.2)	12	8.8
ICD-10 diagnosis M (Musculoskeletal system, mainly)	432 (10)	63	15
ICD-10 diagnoses M and K (Fibromyalgia and IBS)	24 (0.6)	<10	N/A
ICD-10 diagnosis N (Genitourinary system)	156 (3.7)	10	6.4
ICD-10 diagnosis R (Functional tachycardia)	71 (1.7)	<10	N/A
ICD-10 diagnosis T (Subcutaneous emphysema)	83 (1.9)	<10	N/A

ICD-10 – International Classification of Diseases, 10th Revision, IBS – inflammatory bowel disease, N/A – not applicable

*To protect privacy ICD-10 diagnose blocks with less than 10 patients were omitted from the table, and those with less than 10 deaths are not reported in detail.

The most common comorbidity was cardiovascular disease both among IRD and non-IRD patients (20 *vs*. 21%). Up to 98% of non-IRD patients had a comorbid disease, whereas in IRD patients the corresponding proportion was 92%.

### All-cause mortality over the 2-year follow-up period

The mean ± SD follow-up time was 15.2 ± 2.9 months. The crude ACM rate over the 2-year follow-up was higher in IRD patients (16.2%, n = 452 of which 73 died) compared to other patient categories formed on one-character ICD-10 classes (**Table 2**). Concerning specific diagnoses with at least 10 deaths, the crude ACM rate was highest in patients with rheumatic fever (31.9%, n = 47 of which 15 died) and those with antibiotic associated colitis (18.9%, n = 132 of which 25 died). In the latter group, however, the original reason for the antibiotic treatment was unknown. With respect to chronic conditions, arthritis was associated with the highest crude ACM rate (16.3%, n = 301 of which 49 died) followed by osteoporosis (15.5%, n = 129 of which 20 died) and multiple sclerosis (15.3%, n = 118 of which 18 died). For example, the crude ACM rate in patients with coronary heart disease was 11.7% (n = 368 of which 43 died).

### All-cause mortality and inflammatory rheumatic disease

**Figure 1:**
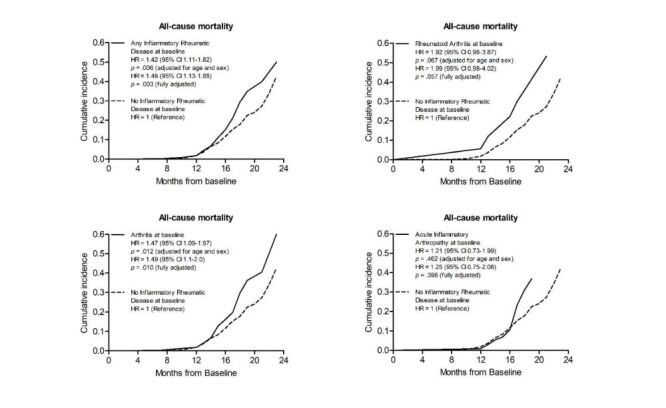
Cumulative incidence of all-cause mortality by Inflammatory Rheumatic Disease status. HR – hazard ratio, CI – confidence interval.

In IRD patients, the 2-year mortality rate per 100 person-years was 13 (95% CI = 10–16), whereas among patients having no IRD, it was 9 (95% CI = 8–10) (**Table 3**). Inflammatory rheumatic diseases increased the hazard of ACM by 1.4 (95% CI = 1.1–1.8, *P* = 0.006) when adjusted for age and sex and by 1.5 (95% CI = 1.1–1.9; *P* = 0.003) when fully adjusted for all baseline characteristics (**Figure 1**, **Table 3**). Concerning the largest IRD diagnostic groups the respective hazard ratios (HR) were as follows: RA = 1.9 when adjusted for age and sex (95% CI = 1.0–3.9; *P* = 0.067) and 2.0 (95% CI = 1.0–4.0; *P* = 0.057) when fully adjusted, arthritis 1.5 when adjusted for age and sex (95% CI = 1.1–2.0; *P* = 0.012) and 1.5 (95% CI = 1.1–2.0; *P* = 0.010) when fully adjusted, and acute inflammatory arthropathy 1.2 when adjusted for age and sex (95% CI = 0.7–2.0;  *P* = 0.462) and 1.2 (95% CI = 0.8–2.1; *P* = 0.396) when fully adjusted (**Figure 1**).

**Table 3:** All-cause mortality rate during the 2-y follow-up period by the inflammatory rheumatic disease status.

Event	No IRD	IRD	*P*-value
n	3821	452	
PY, n	4836.7	574.0	
Events, n	436	73	
Rate per 100 PY (95% CI)	9.01 (8.24 − 9.86)	12.72 (10.26 − 15.76)	
Hazard ratio* (95% CI)	1	1.42 (1.11 − 1.82)	0.006
Hazard ratio† (95% CI)	1	1.46 (1.13 − 1.88)	0.003

CI – confidence interval, IRD – inflammatory rheumatic disease, PY – person-years

*Adjusted for age and sex in the Cox proportional hazards regression model.

†Fully-adjusted with all baseline characteristics.

**Figure 2:**
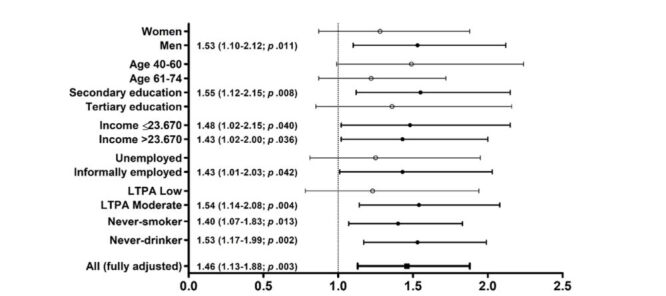
Inflammatory Rheumatic Disease (IRD) *vs*. no IRD hazard ratio (HR) for death in the largest subgroups with n >1000. CI – confidence interval, KES – Kenyan shillings per month, LTPA – Leisure-time physical activity.

Broadly, statistically significant IRD vs no IRD HRs for death were detected in the largest subgroups, such as men, those with secondary education, having an informal job, being moderately physical active during leisure time, and those who had never smoked tobacco or drunk alcohol (**Figure 2**, Table S1 in the **Online Supplementary Document**). There was no evidence of subgroup interactions *i.e*. the subgroup did not affect the association between ACM and IRD but the IRD *vs*. no IRD HR for death was >1 in all subgroups except for ever-drinkers among whom it was <1 and statistically non-significant (S1).

Regarding comorbidities (Table S2 in the **Online Supplementary Document**), the age- and sex-adjusted HR for death in IRD vs non-IRD patients was statistically significant among those with a coexisting disease of the blood or blood-forming organs, mostly an unspecified anaemia (HR = 2.2; 95% CI = 1.0–4.6, *P* = 0.047). Only 107 (2.5%) patients had no comorbidity, and among them IRD did not increase the hazard of death (HR = 0.5; 95% CI = 0.1–2.3, *P* = 0.376).

## DISCUSSION

### Main findings

This prospective follow-up study of 4273 patients from Nairobi metropolitan area showed that IRD increases the hazard of ACM regardless of typical baseline characteristics. Although patients with IRD were older, heavier, and more likely to consume more alcohol compared to non-IRD patients, there was no evidence of subgroup interactions regarding the association between IRD and ACM, but IRD increased the hazard for death in general. Higher risk of death amongst IRD patients has been attributed to severe comorbid conditions, poor functional capacity, and markers of IRD severity, such as erythrocyte sedimentation rate [16]. Additionally, there is overwhelming evidence showing increased mortality caused by persistent inflammation, which is a classic symptom of IRD [17]. In this study, we were unable to attribute the observed mortality to pathophysiological mechanisms due to the absence of cause-specific mortality data.

The 2-year hazard of ACM was particularly high and statistically significant among IRD patients with a coexisting disease of blood or blood-forming organs. Among IRD patients with no comorbidities the hazard of death was not increased compared to patients with no IRD and no comorbidities. The result of this study that IRD may increase the risk of death especially when coexisting with other conditions is in accordance with earlier findings concerning other populations [1,16]. Inflammatory rheumatic disease patients frequently suffer from comorbidities, such as metabolic syndrome including obesity, hypertension, elevated glucose levels, and dyslipidaemia [9]. Mortality in stand-alone IRD cases compared to the general population is reported to have improved over time [18], which could be due to proactive disease management and frequent medical monitoring among IRD population [19].

### Comparison with previous studies

To the best of our knowledge, no prospective follow-up study has addressed ACM among IRD patients in Kenya or neighbouring Eastern Africa countries previously. At large, the main findings of our study were consistent with the results of earlier studies conducted to investigate ACM among IRD patients in Western population, and reporting an increased mortality among IRD patients [1,20]. In this present study, having an IRD increased the hazard for ACM by 1.2–2.0 times depending on the condition. In a study conducted to explore mortality and causes of death among Norwegian patients with RA, psoriatic arthritis (PsA) and axial spondyloarthritis (axSpA), RA and axSpA were associated with an increased risk of ACM (HR = 1.45 (95% CI = 1.41–1.48) and HR = 1.38 (95% CI = 1.28–1.38), respectively [2].

Our findings show that HR for death in IRD was highest in those with a comorbidity of blood or blood-forming organs. Generally, comorbidities have been associated with an increased risk of death among IRD patients [21]. The presence of metabolic syndrome in IRD patients appears to double the risk of cardiovascular diseases, thus exacerbating the risk of death and hence higher mortality in IRD patients with comorbidities [16].

It is important to notice that many previous mortality studies in the present context have focused solely on RA [3,7,18] unlike our study that deals with IRD, a combination of seven conditions (PsA, ankylosing spondylitis, enteropathic arthritis, RA, rheumatic fever without mention of heart involvement, systemic sclerosis, and acute inflammatory arthropathy as a combination of acute inflammatory joint symptoms not aligning with chronic or specific IRDs). The results regarding the IRD diagnostic groups suggested that arthritis, but also RA, were potentially driving the observed mortality patterns, which means that the term ‘IRD’ in this study may refer mainly to arthritis and RA but less evidently to other conditions included.

### Future directions

From a clinical perspective, our findings underscore the urgent need for integrated care models that address both IRD management and comorbidity screening in resource-limited settings like Kenya. Future clinical practice should prioritise early identification and holistic management of comorbidities, particularly haematological disorders, given their significant impact on mortality risk in IRD patients. The development of standardised protocols for regular comorbidity assessment, including cardiovascular risk stratification and metabolic syndrome screening, could substantially improve patient outcomes [22]. Additionally, establishing specialised rheumatology centres with multidisciplinary teams including cardiologists, endocrinologists, and haematologists would facilitate comprehensive care delivery and ensure timely intervention for high-risk patients.

From a health policy standpoint, these results highlight critical gaps in health care infrastructure that require systematic addressing. Policymakers should prioritise the integration of rheumatology services into national health insurance schemes and develop sustainable financing mechanisms for long-term IRD management. The establishment of national registries for IRD patients would enable better disease surveillance, resource allocation, and quality of care monitoring [23]. Furthermore, capacity building through specialised training programmes for health care providers and the development of clinical guidelines adapted to local contexts and resource constraints are essential for improving IRD care delivery across sub-Saharan Africa.

This study concurs with results published in the last recent decades regarding mortality among IRD patients and indicates the need for future follow-up studies in the context, especially in the regions of sub-Saharan Africa. Longitudinal cohort studies with longer follow-up periods are needed to better understand disease progression patterns and identify modifiable risk factors specific to African populations. Future research should also investigate the cost-effectiveness of different treatment strategies and care models to inform evidence-based policy decisions and optimise resource utilisation in lower- and middle-income countries.

### Strengths and limitations

Our study is the first one dealing with the Eastern Africa geographical region and attempting to address a broad spectrum of IRDs. Most studies have focused on mortality in a specific group of IRDs, which may underestimate the overall burden caused by IRDs. This study uses baseline registry data combined with active follow-up data from a health facility covering a large metropolitan area. An important limitation of this study is the use of all-cause mortality without cause-specific death data, which limits our ability to determine the pathophysiological mechanisms underlying the observed mortality differences in IRD patients. While we observed higher mortality rates, particularly among IRD patients with comorbidities, we cannot establish whether deaths were directly related to inflammatory disease processes, treatment complications, or other factors. Future studies with cause-specific mortality data are needed to elucidate the pathways through which IRD influences survival outcomes in sub-Saharan African populations.

As another potential limitation, we acknowledge the diagnostic procedures of IRD applied in the target hospital of this study may not entirely fulfil classification criteria assigned by the American College of Rheumatology and European alliance of Associations for Rheumatology. The potential misclassification due to non-specialist diagnoses, however, should not compromise our main conclusion, as, presumably, patients can be misclassified in both directions; IRD to non-IRD and vice versa.

Additionally, conducting this study in Nairobi's urban hospital setting limits generalisability to rural populations, where poorer health care access and later disease presentation may lead to either higher baseline mortality or different comorbidity patterns that could alter the observed mortality associations or mask IRD-specific effects.

Although our study had a short follow-up period with the mean follow-up time of 15 months, we were able to detect the association between IRD and AMC, which may suggest the association is even stronger than we reported.

Considering the recognised strengths and limitations, we recommend generalising the present results with caution and propose applying them as a reference in future IRD studies in the region of sub-Saharan Africa.

## CONCLUSION

Inflammatory rheumatic disease patients, and especially those with arthritis or RA, may have a higher risk of mortality compared to their characteristic-matched peers with no IRD. The present study revealed the association between IRD and AMC being most obvious in IRD patients having a coexisting disease of blood or blood-forming organs. Inflammatory rheumatic diseases did not increase the hazard of death among patients with no comorbidity compared to patients with no IRD and no comorbidities.

## Additional material


Online Supplementary Document
