# Supplementary material for: All-cause 2-year mortality after hospital discharge among 4273 adults in Nairobi, Kenya with a special focus on inflammatory rheumatic diseases
Source: J Glob Health. 2025 Aug 4;15:04226. doi: 10.7189/jogh.15.04226 (PMC12320198; doi:10.7189/jogh.15.04226)
Supplement: Online Supplementary Document [file jogh-15-04226-s001.pdf]

Supplement to: Onchong'a B, Sokka-Isler T, Mäntyselkä P, Voutilainen A. All-cause 2-year mortality after hospital discharge among 4273 adults in Nairobi, Kenya with a special focus on inflammatory rheumatic diseases. J Glob Health. 2025;15:04226.

Table S1. All-cause mortality rate during the 2-year follow-up period by the subgroup and the inflammatory rheumatic disease status

Subgroup	Total	No IRD	IRD	*p*-value	*p*-value for interaction
Women (*n*; events)	1966; 221	1755; 191	221; 30		Sex .340
	HR	1	1.28 (0.87–1.88)	.209	
Men (*n*; events)	2307; 288	2066; 245	241; 43		
	HR	1	1.53 (1.10–2.12)	.011	
Age 24–39 years (*n*; events)	41; <10	N/A	N/A		Age .532
	HR	1	N/A	N/A	
Age 40–60 years (*n*; events)	1628; 218	1497; 191	131; 27		
	HR	1	1.49 (0.99–2.24)	.055	
Age 61–74 years (*n*; events)	2146; 252	1871; 213	275; 39		
	HR	1	1.22 (0.87–1.72)	.255	
Age 75–83 years (*n*; events)	457; 37	411; 30	46; 7		
	HR	1	2.19 (0.95–5.03)	.065	
Age unknown (*n*; events)	<10; N/A	N/A	N/A		
	HR	1	N/A	N/A	
Primary education (*n*; events)	673; 84	608; 76	65; 8		Education .906
	HR	1	1.11 (0.54–2.31)	.774	
Secondary education (*n*; events)	2222; 278	1975; 234	247; 44		
	HR	1	1.55 (1.12–2.15)	.008	
Tertiary education (*n*; events)	1334; 143	1203; 122	131; 21		
	HR	1	1.36 (0.85–2.16)	.198	
Education unknown (*n*; events)	44; <10	N/A	N/A		
	HR	1	N/A	N/A	

IRD: inflammatory rheumatic disease; KES: Kenyan shilling; PAL: physical activity level; HR: hazard ratios (95% confidence intervals) were adjusted for age and sex in the Cox proportional hazards model; N/A: not applicable.

Interactions were considered as multiplicative interactions and adjusted for age and sex. To protect privacy subgroups with less than 10 patients and/or deaths are not reported in detail.

Table S1. Continues

Subgroup	Total	No IRD	IRD	*p*-value	*p*-value for interaction
Income ≤23,670 KES/month (*n*; events)	1647; 204	1460; 171	187; 33		Income .861
	HR	1	1.48 (1.02–2.15)	.040	
Income 23,671–119,999 KES/month (*n*; events)	2526; 292	2270; 252	256; 40		
	HR	1	1.43 (1.02–2.00)	.036	
Income unknown (*n*; events)	100; 13	91; 13	9; 0		
	HR	1	N/A	N/A	
Unemployed (*n*; events)	1417; 171	1265; 148	152; 23		Employment .431
	HR	1	1.25 (0.81–1.95)	.318	
Informally employed (*n*; events)	1973; 241	1756; 203	217; 38		
	HR	1	1.43 (1.01–2.03)	.042	
Formally employed (*n*; events)	836; 91	755; 79	81; 12		
	HR	1	1.74 (0.94–3.21)	.077	
Employment status unknown (*n*; events)	47; <10	N/A	N/A		
	HR	1	N/A	N/A	
PAL low (*n*; events)	1162; 150	1025; 128	137; 22		PAL .422
	HR	1	1.23 (0.78–1.94)	.367	
PAL moderate (*n*; events)	3051; 353	2744; 302	307; 51		
	HR	1	1.54 (1.14–2.08)	.004	
PAL high (*n*; events)	10; <10	N/A	N/A		
	HR	1	N/A	N/A	
PAL unknown (*n*; events)	50; <10	N/A	N/A		
	HR	1	N/A	N/A	

IRD: inflammatory rheumatic disease; KES: Kenyan shilling; PAL: physical activity level; HR: hazard ratios (95% confidence intervals) were adjusted for age and sex in the Cox proportional hazards model; N/A: not applicable.
Interactions were considered as multiplicative interactions and adjusted for age and sex. To protect privacy subgroups with less than 10 patients and/or deaths are not reported in detail.

Table S1. Continues

Subgroup	Total	No IRD	IRD	*p*-value	*p*-value for interaction
Ever-smoker (*n*; events)	472; 57	414; 48	58; 9		Smoking .921
	HR	1	1.65 (0.80–3.39)	.174	
Never-smoker (*n*; events)	3801; 452	3407; 388	394; 64		
	HR	1	1.40 (1.07−1.83)	.013	
Ever-drinker (*n*; events)	445; 58	369; 49	76; 9		Drinking .143
	HR	1	0.87 (0.42−1.78)	.694	
Never-drinker (*n*; events)	3828; 451	3452; 387	376; 64		
	HR	1	1.53 (1.17−1.99)	.002	

IRD: inflammatory rheumatic disease; KES: Kenyan shilling; PAL: physical activity level; HR: hazard ratios (95% confidence intervals) were adjusted for age and sex in the Cox proportional hazards model; N/A: not applicable.
Interactions were considered as multiplicative interactions and adjusted for age and sex. To protect privacy subgroups with less than 10 patients and/or deaths are not reported in detail.

Table S2. All-cause mortality rate during the 2-year follow-up period by the main comorbidity and the inflammatory rheumatic disease status

Comorbidity	Total	No IRD	IRD	*p*-value
No comorbidities (*n*; events; HR)	107; 12	1	0.50 (0.11–2.33)	.376
ICD-10 diagnosis A (*n*; events; HR)	221; 26	1	1.14 (0.37–3.45)	.821
ICD-10 diagnosis B (*n*; events; HR)	299; 45	1	1.67 (0.76–3.68)	.202
ICD-10 diagnosis C (*n*; events; HR)	52; 13	1	1.12 (0.23–5.55)	.890
ICD-10 diagnosis D (*n*; events; HR)	538; 56	1	2.15 (1.01–4.58)	.047
ICD-10 diagnosis E (*n*; events; HR)	452; 50	1	2.14 (0.99–4.65)	.054
ICD-10 diagnosis G (*n*; events; HR)	213; 30	1	1.38 (0.41–4.63)	.607
ICD-10 diagnosis I (*n*; events; HR)	893; 102	1	1.70 (0.98–2.95)	.061
ICD-10 diagnosis J (*n*; events; HR)	269; 29	1	2.48 (0.93–6.63)	.070
ICD-10 diagnosis K (*n*; events; HR)	222; 25	1	0.34 (0.04–2.56)	.294
ICD-10 diagnosis N (*n*; events; HR)	319; 32	1	0.79 (0.24–2.63)	.700
ICD-10 diagnosis R (*n*; events; HR)	522; 60	1	1.81 (0.88–3.69)	.105
ICD-10 diagnosis S (*n*; events; HR)	88; 17	1	1.07 (0.29–3.91)	.919

IRD: inflammatory rheumatic disease; HR: hazard ratios (95% confidence intervals) were adjusted for age and sex in the Cox proportional hazards model; ICD-10: International Classification of Diseases 10th Revision.
ICD-10 diagnose blocks with less than 10 deaths were omitted from the table to protect privacy.
